# Global Policy to Reduce the Incidence of Infection Spreading in Non-Vaccinated Healthcare Workers: A Literature Review

**DOI:** 10.3390/vaccines10122058

**Published:** 2022-11-30

**Authors:** Cristiana Ferrari, Giuseppina Somma, Lorenzo Ippoliti, Andrea Magrini, Luca Di Giampaolo, Luca Coppeta

**Affiliations:** 1 Department of Occupational Medicine, University of Rome “Tor Vergata”, 00133 Rome, Italy; 2 Department of Occupational Medicine, University of Chieti “G. D’Annunzio”, 66100 Chieti, Italy

**Keywords:** SARS-CoV-2, healthcare workers, global policy, COVID-19, non-vaccinated HCWs

## Abstract

Healthcare workers (HCWs) are at increased risk of SARS-CoV-2 infection because of their occupational exposure. Moreover, they can be a vehicle for the virus transmission among patients. The vaccination of healthcare personnel against COVID-19 is crucial in fighting the spread of SARS-CoV-2 infection, together with strict sanitary procedures that aim to limit the risk of contagion. Unfortunately, even if COVID-19 vaccination has been proved one of the most effective tools for protecting against COVID-19, many healthcare professionals are not yet vaccinated. The aim of the current review is to contribute to identifying an effective strategy for COVID-19 prevention especially among non-vaccinated HCWs. In this review, we collected the most recent and relevant findings from literature on the protection of unvaccinated HCWs, identifying three types of measures as principal actions to protect those operators: addressing vaccine hesitancy, improving non-pharmaceutical interventions and promoting actions at personal level (respiratory hygiene, hand hygiene and use of PPE). All these interventions are very effective in preventing contagion, if well respected and conducted; nevertheless, it is essential to promote vaccination, as it is the most effective measure.

## 1. Background

Susceptible healthcare workers (HCWs) have a higher risk of being infected and can be vehicles for the evolution of outbreaks in healthcare settings. HCWs vaccination is very important not only to protect them from occupational exposure, but also to protect their patients [1].

As of 25 December 2021, more than 79% of all adults (>18 years) in the European area and over 72% of all adults (>18 years) in the USA received a complete vaccination schedule against COVID-19 [2,3]. Since the first authorization for emergency use for the COVID-19 vaccine, priority has been given to healthcare professionals [4].

Healthcare professionals are both a risk-exposed population group and a vehicle for transmitting the virus to patients. 

They also have a significant impact on the population’s decisions on vaccination and can influence patients’ perceptions of the issue [5,6,7].

Vaccination is a crucial step in the fight against infectious disease. The large-scale adoption of childhood vaccinations has been part of the best public health policies, to control or eliminate multiple infectious diseases [8].

Despite the evidence on the efficacy and safety of vaccination, many healthcare professionals are not vaccinated or fully vaccinated yet. Unfortunately, vaccine hesitancy, delay in vaccine acceptance and rejection are increasing phenomena among HCWs and the general population [9,10]. For these reasons, it is very important to investigate global policies on how to defend the health of unvaccinated operators. It is also very important to reflect on the possibility of a reduction in the effectiveness of the vaccine over time as has been observed for other vaccine-preventable infectious diseases, such as mumps [11,12], measles [13,14] and rubella [15]. Therefore, it is important to foresee future strategies to limit the contagion.

The National Institute for Occupational Safety and Health (NIOSH) recommends a hierarchy of controls to determine and adopt effective and achievable solutions [16]. The most difficult methods to apply in an existing system are elimination and replacement, although they are the most effective in reducing the risk. Technical controls are preferable to personal protective equipment (PPE) to control exposure in the workplace, as they are designed to eliminate the risk at its source, before it encounters the worker. Administrative controls and the use of PPE are associated with other existing programs where risks are not well controlled. They can be inexpensive to start up, but costly when used in the long run.

It has been reported that the use of masks by healthcare professionals during a respiratory disease pandemic can minimize the spread of the infection and its economic impact, if masks are used correctly and consistently, providing an effective non-pharmaceutical intervention in controlling the disease [17]. Moreover, our study on the diffusion of SARS-CoV-2 in a hospital environment shows the importance of using masks in the prevention of contagion, and that in assessing the risk of contacts their use is more significant than the distance and duration of contact [18].

The objective of this review is to investigate the global strategies and protocols adopted for the protection of unvaccinated health workers.

## 2. Methods

In this literature review, we collected the most recent and relevant findings from literature such as original articles, systematic reviews and statements from government health agencies and occupational health agencies regarding global policies on the protection of unvaccinated healthcare workers.

Inclusion criteria: studies (systematic reviews: meta-analyses) that evaluated the use of face masks compared to other types of masks or the non-use of masks in the health of non-vaccinated healthcare workers for the prevention of respiratory viral infections were considered eligible for inclusion in this rapid review. No filters were applied related to date, language or publication status. Laboratory studies were included in this quick review to verify the effectiveness of incorporating this type of material for droplet containment in non-vaccinated healthcare workers. All studies evaluated the effectiveness of global policies on how to protect non-vaccinated healthcare workers. The results indicate superiority for the policies indicated for use by health professionals and verify that, depending on the type of policies, even with lesser efficacy, they are capable of containing more than 90% of the droplets. 

Exclusion criteria: studies that evaluated health professionals, global policies, as well as preclinical, health technology assessment studies, editorials, comments, opinion articles and narrative reviews were excluded.

Information sources and search strategies: literature searches were performed in Medline (via Pubmed), Scopus and Cinahl, and The Cochrane Library databases. The search strategies used free descriptors and terms for the words “Coronavirus”, “COVID-19, “Severe acute respiratory syndrome” and “healthcare staffs”. For the complementary search, Google Scholar was used. The search was conducted on November 8, 2021.

## 3. Results

USA’s Occupational Safety and Health Administration has produced a guide to support employers for protecting unvaccinated employees [19]. This document suggests 11 interventions that can be adopted to protect workers who are unvaccinated and therefore at risk, and thereby to reduce the diffusion of SARS-CoV-2.

UK’s NHS has published a document called “Guidance to support COVID-19 vaccine uptake in frontline staff: Guidance for HR directors” [20], which lists important measures for unvaccinated staff. These guidelines include: the appropriate use of PPE, make sure workers are familiar with infection control basic rules and are committed to adequate training, have an updated risk assessment to recognize their personal risks, knowledge of the most recent government and professional agency advice, the possibility of relocating to an environment less subject to exposure.

Finally, three types of interventions have been identified as main actions aimed at protecting the health of the unvaccinated operators: addressing vaccine hesitancy, improving non-pharmaceutical interventions and promoting actions at personal level (respiratory hygiene, hand hygiene and use of PPE).

### 3.1. Addressing Vaccine Hesitancy

Although patients consider HCWs as a standard-setters regarding health issues, many current studies show that a significant rate of such operators, including those who directly provide vaccination, are hesitant for themselves, their relatives and patients. Most of these findings in the past were focused on the acceptance of influenza vaccines whereas in the last months a growing body of evidence is emerging regarding COVID-19 vaccine.

Many of these studies found that HCWs have not been vaccinated against the flu because they did not have time [21,22]; considered themselves not at risk of influenza [23,24]; felt fit or did not receive the ordered vaccine [25,26], or had doubts regarding the efficacy and safety of the vaccine [27,28]. 

The amount of hesitant European HCWs is unknown but some studies reported that many operators have doubts regarding vaccines, so they may not recommend them. A recent study pointed out that about 16–43% of family doctors in France have reported not recommending their patients to get vaccinated, despite vaccination being acknowledged as the most effective policy to defeat infectious disease by all government health agencies and occupational health agencies examined [29]. Around the world, all healthcare professionals have been strongly advised to get vaccinated against COVID-19.

The European Centre for Disease Prevention and Control (ECDC) has produced a document for public health authorities, both regional and national, risk communication specialists and policy makers in European countries, promoting the use of the 5C model in regions where acceptance is difficult and recommending the adoption of vaccination against SARS-CoV-2 and the design and implementation of interventions to promote vaccine acceptance [30]. According to the Strategic Advisory Group of Experts (SAGE) on immunisation, the vaccine hesitancy is “a behaviour, influenced by a number of factors including issues of confidence (level of trust in vaccine or provider), complacency (do not perceive a need for a vaccine, do not value the vaccine), and convenience (access issues)” [31]. Hesitant subjects are a miscellaneous group with various levels of doubts on vaccination: there are persons who complete reject vaccination, others who reject or delay a determined type of vaccine, and others who fear vaccination but accept all vaccines [32,33]. Policies to improve vaccine uptake were: better information, more regulations by health authorities, open communication between physician and patient, and training for operators to develop the ability to address patient hesitancy.

Given the many concerns related to vaccine hesitation, more comprehensive and specific action is needed. Although most current interventions focus on informing about the vaccine efficacy and safety, doubts, pointed out in various studies, identify other determinants of hesitation.

### 3.2. Non-Pharmaceutical Interventions

According to ECDC guidelines to adjust non-pharmaceutical interventions according to vaccination status, the following interventions have been planned to reduce community contagion during the COVID-19 pandemic [34]: most importantly, the physical distancing in various settings; the promotion of self-isolation of subjects with COVID-19 suggestive symptoms; the simplification of access to diagnostic tests with the consequent fast contact tracing and the start of quarantine of contacts. Other useful interventions are: to promote the “social bubble” (contact with same people); to avoid, cancel, or postpone crowded meetings (both indoor and outdoor), promoting webinars; to encourage smart working activities; to close businesses where social distancing cannot be respected. Lastly, it is important to adopt environmental measures, e.g., to sanitize and disinfect the frequently touched surfaces, and to ensure appropriate air changes in closed spaces to decrease the risk of virus transmission. All the above-mentioned interventions assume relevant significance in health care environments to lower the nosocomial risk of infection of the operators.

All countries have adopted interventions to limit the virus spread, from simple recommendations to avoid gatherings up to generalized lockdowns. 

To contain the infection from COVID-19, the Italian legislation recommends the application of some basic rules: wash frequently hands with soap and water or with hydro-alcoholic solutions; avoid close contact with persons affected by acute respiratory infective disease; avoid handshakes and hugs; keep one meter of distance from other people; practice respiratory hygiene (cover mouth and nose or use a handkerchief to sneeze or cough and avoid touching respiratory secretions); avoid the mixed use of glasses or bottles, especially in physical activity; avoid touch the face (eyes, nose and mouth) with the hand; avoid use antiviral of drugs or antibiotics, if not ordered by a doctor; use alcohol based disinfectants or chlorine to sanitize surfaces.

It Is heavily recommended to adopt respiratory tract protections as a further sanitary and personal protective measure, in all social contacts.

A research study conducted in 131 countries measured the impact of non-pharmacological measures, and their subsequent removal, on the spread of the infection during the first epidemic wave, estimated by the reproduction number Rt. The Rt number indicates the number of people on average infected by each positive case in the unit of time considered [35]. Depending on the interventions adopted (e.g., the prohibition of public events), the Rt number decreased between 3% and 24% after 28 days from their respective introduction. Conversely, the removal of the measures brought to an increase in the Rt number of between 11% and 25% after 28 days from the end of the restrictions, with more significant increases after the reopening of the schools and the revocation of the prohibition of gatherings of more than ten people. The study finally has demonstrated that there is a variable delay between one and three weeks between the introduction of measures and the effect on the Rt number, and an even wider time interval for the increase in the Rt value after the abolition of the restrictions.

A study carried out by INAIL, the Istituto Superiore di Sanità (ISS, the National Institute of Health in Italy) and the Bruno Kessler Foundation, estimated that the measures adopted in Italy during the second epidemic wave of autumn 2020, based on the subdivision of the territory into bands of different colours (according to the epidemiological indicators), have reduced hospitalizations by more than a third in the period between 6 and 25 November. In that period the total number of hospitalizations for COVID-19 in medical areas rose from about 23,000 to over 34,000, and that of those hospitalized in intensive care from about 2400 to over 3800 [36].

### 3.3. Promoting Action at Individual Level 

As SARS-CoV-2 spreads by droplets, it is important to cover the mouth and nose with a paper tissue or handkerchief when sneezing and coughing in order to reduce person-to-person transmission. Moreover, used tissues have to be accurately disposed of and hands have to be washed with soap and water [37].

Compliance with all respiratory hygiene measures is a good practice to be implemented to prevent all diseases that can be spread by air and direct contact with respiratory secretions, such as COVID-19.

Hygiene of hands is a rational recommendation, with low costs and no risk. In combination with other measures, such as face masks, its effectiveness will likely increase.

As for hand hygiene, this refers to repeated and adequate washing with soap and water or the use of handkerchiefs, gel or solutions. Hands should be washed thoroughly with soap and water for about 40 s; if hands are dirty, soap and water should be used before disinfectants [38]. 

Alcohol-based hand sanitizers do not offer, in community settings, a great additional advantage over water and soap and, if used, they should contain at least 60–85% alcohol [17,37]. 

The importance of personal protective equipment (PPE) to protect HCWS and patients from risk of infection is globally known. The use of PPE has been fundamental to save life during epidemics of Ebola and Zika and influenza pandemics.

In order to prevent facial and conjunctival contamination, there are many respiratory PPE, such as masks, face shields/visors, and respirators [39]. PPE has a crucial role reducing COVID-19 spread both in the hospital and community settings. In hospital settings in in the bronchoalveolar specimens and in the sputum of SARS-CoV-2 infected patients there is a major viral load and nurses are at higher risk to contract the infection, in the absence of PPE, especially during patient intubation procedures [40], and others [41].

There are two kinds of respiratory protection equipment available: the medical grade facemasks and the filtering-facepiece respirators [42]. 

Facemasks have been included in the universal precautions in clinical settings, but they are inadequate for HCWs in particular circumstances [43,44]. 

The main type of the facemask, approved for HCWs, is the facepiece N95 respirators, NIOSH (National Institute for Occupational Safety and Health) with recommended filtering [45]. This mask can trap 95% of particles larger than 0.3 µm, under the airflow rate of 85 L/min, the rate of a difficult and deep breath [46]. 

Masks’ filtering ability depends on the dimension and trajectory of the particles, with small floating aerosols more difficult to filter than larger moving particles [47].

The N95 mask is functionally efficient and may also have a good face-fit [48].

In a recent study, it was found that people who received a regular daily supply of new surgical masks had a lower risk of rhinovirus infection than those who received a limited supply of cloth masks. [49].

Data published by the CDC show how the use of masks—both cloth and surgical—can significantly reduce the transmission of the virus, up to over 95%, if worn correctly. According to the CDC, the optimal solution is a surgical mask (or medical) tightly knotted around the ears, or a double mask, surgical and cloth [50].

Literature data suggest that when compliance is high, using masks is more effective in reducing the viral diffusion. It could be useful that health authorities supply clear indications for the use, reuse, sanitation and production of face masks and consider their dispensation when the availability allows [51].

Regarding unvaccinated operators, the UK National Health Service has published a guide for employers in the healthcare sector in England called “Vaccination as a Condition of Deployment (VCOD) for Healthcare Workers” (6 December 2021, Version 1, UK NHS). In this document, it is recommended to redeploy health workers who do not get vaccinated. This advice was related to a growth in vaccine uptake by 10%.

On the other hand, there is a minority of health workers who cannot be vaccinated for health or religious reasons. Different characteristics of unvaccinated subjects were shown to be associated with a greater risk of becoming infected by SARS-CoV-2. [52].

We found several government health agency documents that contain recommendations in this regard [53].

## 4. Discussion

This review found two different documents indicating measures or interventions for unvaccinated healthcare workers. In all the documents analyzed, direct communication with unvaccinated staff is reiterated to ensure correct formation about the risks of the disease and vaccination efficacy. Publishing time should have affected the outcomes of the above-mentioned studies, since the COVID-19 situation has dramatically changed over time and operators believe as well that risk perception may have changed during the last two years [54].

Possible reasons for low vaccine uptake among HCWs have been reported in the literature: poor attitude to referring to evidence-based sources of information on vaccines, lack of confidence in government and scientific community policy, belief in conspiracy theories including the unwavering conviction of secret agreements between pharmaceutical companies and the government [55,56].

Mandatory SARS-CoV-2 vaccination policies have been introduced for HCWs in many countries, including in Italy with the Law Decree, No. 44 (1 April 2021). The results of these policies have to be evaluated in further studies, as mandatory vaccination can improve vaccine acceptance, but it could reduce confidence in healthcare organizations and immunization programs [57,58]. Action to improve operators’ trust in institutions and vaccine safety could result in a wider acceptance rate among hesitant subjects.

All government health agencies have created FAQ to inform and train the population on the importance of vaccination, its effectiveness and its safety, for example, the website of the Italian Ministry of Health [53]. Several countries in Europe have imposed mandatory vaccination for health care workers (such as Italy [59], France [60] and Greece [61]).

Mandatory vaccination policies have always raised considerable controversy, but it is essential that healthcare professionals are immune, also for moral reasons, especially for who work in contexts with high-risk patients [62].

The European Court of Human Rights (ECHR) has held that mandating vaccines does not violate human rights law (April 8, 2021) [63].

However, it is important that political decisions do not discriminate against those who cannot have the vaccine due to medical or other reasons (such as pregnancy or religious reasons). The reduction in risks of severe SARS-CoV-2 infection in a target population are related to the vaccine acceptance and rate of vaccine coverage and are influenced by many other factors including age, variants of concern, individual susceptibility, vaccine characteristics and the epidemiological situation [64].

Non-pharmaceutical interventions had an important effect on reducing transmission. These interventions, which are different among countries, included social distancing (such as banning large gatherings), border closures, school closures, measures to isolate symptomatic individuals and their contacts, and large-scale lockdowns of populations with all but essential internal travel banned.

Many studies have analyzed the effects of non-pharmaceutical interventions on the spread of SARS-CoV-2 [65,66,67,68,69,70], and have estimated the reproduction number, which is different among countries [71] and is influenced by economic and social factors [72,73].

Data from the literature clearly demonstrate that early relaxation of safety non-pharmacological interventions can compromise the success of the vaccination campaign by permitting the emergence of variants of concern, favoring the spread among susceptible unvaccinated subjects. To face the diffusion of SARS-CoV-2, the governments have issued warnings and recommendations regarding the adoption of action at individual level and then they have imposed legal restrictions such as lock-down. At the initial phase of the epidemic, people adopted the recommendations weakly, then when it became a pandemic, many subjects still continued to not strictly follow the suggestions. This lack of respect is due to the difficulty in changing people’s behavior [74]. Theoretical models [75,76] have shown that people will only comply with health warnings for:

- Perceived susceptibility.—This refers to a person’s subjective perception of the risk of acquiring an illness or disease. There is wide variation in a person’s feelings of personal vulnerability to an illness or disease.
- Perceived severity.—This refers to a person’s feelings on the seriousness of contracting an illness or disease (or leaving the illness or disease untreated). There is wide variation in a person’s feelings of severity, and often a person considers the medical consequences (e.g., death, disability) and social consequences (e.g., family life, social relationships) when evaluating the severity.
- Perceived benefits.—This refers to a person’s perception of the effectiveness of various actions available to reduce the threat of illness or disease (or to cure illness or disease). The course of action a person takes in preventing (or curing) illness or disease relies on consideration and evaluation of both perceived susceptibility and perceived benefit, such that the person would accept the recommended health action if it was perceived as beneficial.
- Perceived barriers.—This refers to a person’s feelings on the obstacles to performing a recommended health action. There is wide variation in a person’s feelings of barriers, or impediments, which lead to a cost/benefit analysis. The person weighs the effectiveness of the actions against the perceptions that it may be expensive, dangerous (e.g., side effects), unpleasant (e.g., painful), time-consuming, or inconvenient.
- Cue to action.—This is the stimulus needed to trigger the decision-making process to accept a recommended health action. These cues can be internal (e.g., chest pains, wheezing, etc.) or external (e.g., advice from others, illness of family member, newspaper article).
- Self-efficacy.—This refers to the level of a person’s confidence in his or her ability to successfully perform a behavior. This construct was added to the model most recently in mid-1980. Self-efficacy is a construct in many behavioral theories as it directly relates to whether a person performs the desired behavior.

Many of the measures taken to prevent people from being infected require a change of attitude. The use of masks and gloves, proper hand washing and social distancing are all forms of human behavior. Health promoters and health professionals have expertise in health attitudes, so their suggestions and opinions can help governments to achieve the required behavior [74].

The literature includes much evidence showing an optimal SARS-CoV-2 vaccine efficacy and safety and supports the correlation between using the vaccine and COVID-19 prevention [77]. In particular, in vaccinated subjects, evidence highlights a relevant decrease in contagiousness, a lower viral load in the nasal mucosa, and an efficacy in preventing infection of 90% 14 days after the second dose and 80% 14 days after the first dose [78,79]. Interpretation of these results shows the correlation between decrease in SARS-CoV-2 viral load and probability of infection of vaccinated subjects. The molecular hypotheses explain that in the vaccinated and COVID-19 positive subjects the intact virus may be present, but is covered by antibodies that do not allow it to infect other individuals [80,81]. Therefore, vaccination remains the best method to prevent contagion and SARS-CoV-2 diffusion.

## 5. Conclusions

The global strategies and protocols adopted for the protection of unvaccinated health workers can be articulated in three type of interventions: addressing vaccine hesitancy, improving non-pharmaceutical interventions and promoting actions at personal level (respiratory hygiene, hand hygiene and use of PPE). All these measures, if respected and carried out correctly, are very effective in preventing contagion. It is very important to apply these rules in all contexts, not only in the hospital environment, but also in all public places, respecting the rules for one’s own safety and that of others.

Not to discriminate against unvaccinated subjects is a right decision, but it is still essential to promote vaccination, as it is the most effective measure, combined with all the other measures available, to protect yourself and reduce the possibility of developing COVID-19.

## Data Availability

Not applicable.
